# Smoking, health and social justice

**DOI:** 10.1016/j.clinme.2026.100622

**Published:** 2026-07-12

**Authors:** Sanjay Agrawal

**Affiliations:** University Hospitals of Leicester NHS Trust, United Kingdom

**Keywords:** Smoking, Tobacco, Social justice, Opt-out, Health-inequality

## Abstract

**Background:**

National smoking prevalence is declining and new legislation, such as the Tobacco and Vapes Act, will reduce the number of people who take up smoking. However, there are profound disparities in the prevalence of people who currently smoke in the population, and further measures are required to prevent and treat tobacco-related inequality.

**Methods:**

The recently published RCP report '*Smoking, health and social justice* outlines the drivers of smoking inequality, significant gaps in national data collection, the economic impact of smoking and measures to mitigate this wholly preventable cause of disadvantage.

**Conclusion:**

By introducing opt-out treatment of tobacco dependency across the NHS, raising the price of tobacco and applying cross-governmental actions to health-harming industries, the persistent gap in smoking-related inequality can be reduced.

The Tobacco and Vapes Act became UK legislation on 29 April 2026, representing a landmark moment.^1^ As a consequence, people born after 1 January 2009 cannot be legally sold tobacco in the UK, preventing a life blighted by tobacco-induced ill health and premature mortality that continues to affect over a billion people around the world.^2^ In addition, the legislation will provide the UK government with powers to limit the availability, appearance, promotion, outdoor use and product specifications for all tobacco- and nicotine-containing products, including vapes, which have become popular among children, young people and some adults who have never smoked in the UK.^3^

While the legislation is welcome, there is a risk that millions of people still caught in the trap of tobacco addiction will be neglected as policymakers consider ‘the tobacco problem’ to be solved and take forward other policy priorities. Although average UK smoking prevalence has fallen steadily and is currently 10.6%, digging beneath this average uncovers large disparities in smoking prevalence between population groups (see Fig. 1), for example in people with lower educational attainment, people who perform routine and manual work or people living in social housing.^4^

**Fig. 1:**
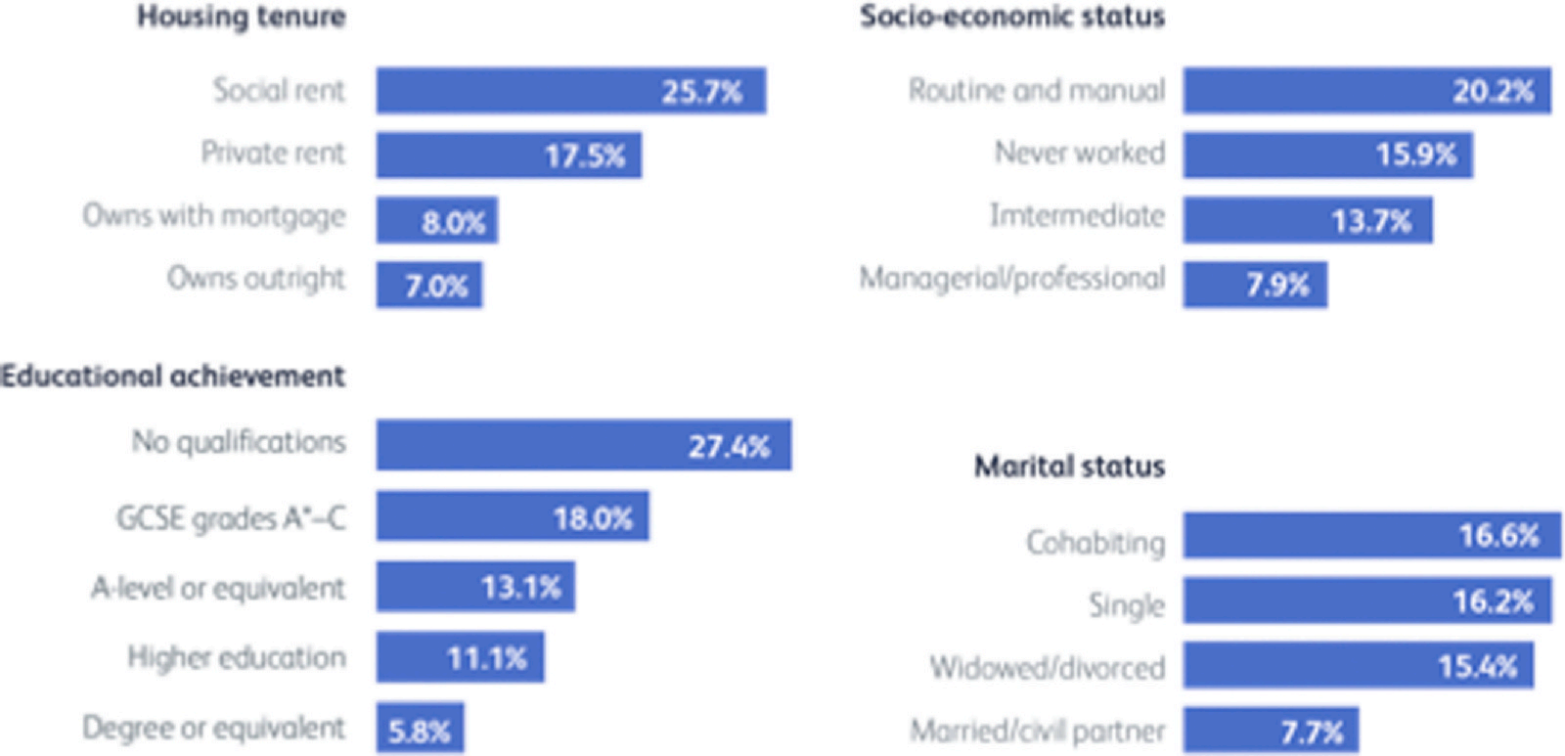
Characteristics of people who currently smoke cigarettes in the UK.^5^

Source: ONS, Smoking habits in the UK and its constituent countries Tables 7, 8, 9, and 13.

These disparities between the least and most advantaged population groups have persisted over many decades, despite the overall drop in smoking prevalence in all four UK nations,^6^ suggesting that they are the result of unequal or unfair distribution of wealth, opportunity and resources, often referred to as ‘social injustice’. The concept of social justice and health inequalities is neither new nor unique to the UK and has been reported for several decades, with reviews by Sir Michael Marmot providing a significant and ongoing call to action for sequential governments.^7^, ^8^

The Royal College of Physicians recently published a comprehensive review of the literature to examine the relationship of smoking and social justice.^9^

First, they consider the wider determinants of health – factors that people do not directly control yet determine where they are born, grow, live, eat, learn, work and age. Numerous factors intertwine and can result in poverty, poor diet, limited attainment and, through cumulative stress, can shape the use of alcohol, tobacco or unhealthy foods. Commercial actors such as transnational tobacco companies actively contribute to drivers of inequality using price policies to attract and retain less advantaged populations and target promotion of their products through sponsorship and social media channels to specific population subgroups. Studies presented suggest a clear dose–response effect – as social risk factors accumulate for individuals (unemployment, poverty, low educational attainment, disability, serious psychological distress and heavy alcohol use), the greater likelihood that they smoke tobacco.^10^ The intersection of multiple conditions, such as mental health disorder and homelessness, exacerbate the use of products such as tobacco and alcohol. The health consequences of using more than one harmful product is multiplicative rather than additive and concentrated in the least advantaged people and communities, exacerbating poverty, poor housing and employment.^11^

Secondly, the report describes an estimated ‘hidden population’ of over a million people who smoke in the UK but are not captured in national surveys and datasets.^12^ The largest group of this hidden population are predominantly young adults who reside temporarily between friends or family members. Other groups in this ‘hidden population’ include people who stay in residential facilities such as student halls of residence, care homes, prisons and detention centres. All of these populations have much higher smoking prevalence than the national average. Further, people who use oral tobacco products or water pipe tobacco users (shisha), predominantly of South-East Asian origin, are largely excluded from national surveys, exacerbating unmet demand for tobacco dependency treatment services and widening the equity gap.^13^ The paucity of national data collection on the use of illicit tobacco used more often in less advantaged communities is exposed.

The third main area of the report focuses on the economic impact of tobacco use, with estimates of the costs of untreated tobacco addiction to individuals, families and society. The total annual cost of smoking to UK public services exceeded £19 billion in 2024, with disadvantaged groups accounting for a disproportionate share of these costs due to higher smoking prevalence. An estimated additional 560,000 households live in poverty due to tobacco addiction, creating a vicious cycle of poverty, stress, smoking and inequity. The report estimates that current funding of treatment (£180 million) for tobacco addiction represents <1% of the costs of smoking to society (£19 billion). Importantly the authors estimated the ‘smokefree dividend’ to society of £10.9 billion if smoking rates were zero, money that would be retained within communities, supporting families, dwarfing the £7 billion tax receipts from tobacco products.^14^

Fourth, more than 30 recommendations and a call to action for cross-governmental policies and NHS interventions are put forward to reduce the persistent health inequality gap and tobacco use (Box 1); the UK should adopt a coherent cross-government approach to counter health-harming industries that shape the availability, affordability and appeal of tobacco and other harmful products; opt-out tobacco dependency treatment for hospital inpatients has been successful in helping more people to quit smoking in less advantaged groups and should be extended to all NHS settings;^15^ national tobacco control policy should be rebalanced towards high-prevalence, underserved groups, investing in better data collation that captures hidden populations to enable targeted action; and regulation of tobacco products should be strengthened – specifically pricing, retail availability and marketing to reduce the impact on less advantaged populations.

Box 1 **Over-arching recommendations and a ‘call to action’.**

**Table:** 

**Recommendation**	**Rationale**
National tobacco control policy should be rebalanced towards high-prevalence, underserved groups	Tobacco-related health inequalities will be reduced if tobacco control policies differentially affect higher prevalence groups such as those living in temporary accommodation, people with mental health disorders, LGBQT communities, unemployed, low-paid workers^9^
The UK should adopt a cross-government approach to counter health-harming industries to better regulate tobacco product pricing, retail availability and marketing to reduce the impact on less advantaged populations	The commercial sector contributes to health inequalities through commercial strategies to make their products easily available, appealing and less expensive, sustaining addiction among vulnerable communities. Several government departments, eg health, local government and treasury, are required to work together to mitigate these commercial drivers of inequality
Opt-out tobacco dependency treatment services should be extended to all NHS settings	Treatments for tobacco dependency are highly cost-effective but rarely delivered. Systematic opt-out provision in NHS inpatient settings has demonstrated much greater access and a higher number of quits in less advantaged populations
Invest in better data collation that captures hidden populations to enable targeted action	Current national data collections omit over a million tobacco users, providing a false estimate of national smoking prevalence and the resources required to tackle tobacco-related health inequality

These recommendations may not be considered ‘novel’, as is the case with many effective public health interventions such as vaccination or minimum unit pricing of harmful commodities; however, to succeed with implementation, deliberate action is required. Specifically, government should set out a new national tobacco control plan, as we have had previously, with clear ambition, interventions, funding and accountability, taking up the recommendations outlined above.

In summary, the Tobacco and Vapes Act will go a long way to provide a smoke-free generation for young people living in the UK and is to be welcomed. However, additional policy changes and sustained investment are required if we are to eliminate the unjust burden tobacco places on our communities, especially those who can least afford it.

## CRediT authorship contribution statement

**Sanjay Agrawal:** Writing – review & editing, Writing – original draft, Conceptualization.

## Funding

This research did not receive any specific grant from funding agencies in the public, commercial, or not-for-profit sectors.

## Declaration of Competing Interest

The authors declare that they have no known competing financial interests or personal relationships that could have appeared to influence the work reported in this paper.
